# Reasons for engagement with online tobacco marketing among US adolescents and young adults

**DOI:** 10.18332/tid/99540

**Published:** 2019-01-10

**Authors:** Samir Soneji, Kristin E. Knutzen, Meghan Bridgid Moran

**Affiliations:** 1 The Dartmouth Institute for Health Policy and Clinical Practice, Dartmouth Geisel School of Medicine, Lebanon, United States; 2 Dartmouth-Hitchcock Norris Cotton Cancer Center, Dartmouth Geisel School of Medicine, Lebanon, United States; 3 Department of Health, Behavior and Society, Johns Hopkins Bloomberg School of Public Health, Johns Hopkins University, Baltimore, United States

**Keywords:** online tobacco marketing, tobacco ads, tobacco coupons

## Abstract

**INTRODUCTION:**

Engagement with online tobacco marketing among US adolescents increased from nearly 9% (2013–2014) to 21% (2014–2015). Such engagement increases the risk of tobacco use initiation. Despite the increase in the prevalence of and risks associated with engagement, the reasons why adolescents and young adults engage are not known.

**METHODS:**

A sample of 2619 adolescents (13–17 years) and 2625 young adults (18–24 years) living in the US participated in an online survey in July–August 2017. Engagement with online tobacco marketing was assessed through five forms of engagement (e.g. watched a video online promoting tobacco products). Reasons for engagement were assessed through an open-ended survey question. Prevalence of reasons for engagement was calculated overall, by tobacco use status, and by age group (adolescents and young adults). Multivariable logistic regression models were fit with engagement as the outcome (overall and specific reasons) and sociodemographics (including age, gender, and race/ethnicity) and tobacco use status (non-susceptible and susceptible never tobacco users; ever, but not past 30-day tobacco users; and past 30-day tobacco users) as covariates.

**RESULTS:**

Across all tobacco use statuses, the leading reasons for engagement were curiosity or desire for general knowledge about tobacco products (3.9%); incidental, unintended or forced exposure to tobacco ad (3.8%); and seeking discounts, coupons, incentives, or contests (2.9%). Susceptible never tobacco users were more likely to engage because of curiosity or general knowledge than non-susceptible never tobacco users (adjusted odds ratio, AOR=6.81; p<0.01). Past 30-day tobacco users were more likely to engage because of discounts, coupons, incentives, or contests and product appeal than ever, but not past 30-day tobacco users (AOR=7.10; p<0.01).

**CONCLUSIONS:**

Stricter state and federal regulation of tobacco marketing, specifically tobacco ads and coupons, and stronger self-regulation by social networking sites could reduce youth engagement with online tobacco marketing.

## INTRODUCTION

Since the 1998 Master Settlement Agreement, internet-based tobacco marketing has grown in prominence, in part because it is less heavily regulated than traditional tobacco marketing^1-3^. Internet-based marketing enables companies to engage with current and potential customers through tailored promotional marketing activities to which consumers are exposed as they spend time online, either through a computer, laptop, or mobile device^1,4–8^. Among tobacco companies, online marketing entails sponsored activities such as establishing and updating promotional websites and social media accounts for products, sending consumers emails about products, and generating publicity for products through sponsored press or videos^9^. Consumers may be passively exposed to such marketing (e.g. by seeing someone re-post a social media post from a tobacco company), but consumers can also more actively engage with such marketing. For example, consumers can explore different pages of a tobacco brand website, actively provide information to a tobacco company in order to receive emails, or like or re-post a tobacco company post from a social media account. In addition to tobacco brand marketing, consumers can also engage with tobacco-related user-generated content on social media sites.

The prevalence of engagement with online tobacco marketing among adolescents increased from 8.7% (2013–2014) to 20.9% (2014–2015) in the US^10^. Engagement with online tobacco marketing is problematic because it increases the risk of tobacco use initiation among never tobacco users and decreases the risk of tobacco use cessation among current tobacco users^11^. Although it is known that engagement with online tobacco marketing is longitudinally associated with tobacco use behaviors and that the level of this risk factor has increased over time^10,11^, it is not known why adolescents and young adults engage. This study addresses this research gap by ascertaining reasons for engagement among a national sample of adolescents and young adults. Knowledge of these reasons could help refine tobacco marketing regulation to reduce engagement with online tobacco marketing.

## METHODS

### Data

A sample of 2619 adolescents (13–17 years) and 2625 young adults (18–24 years) participated in an online survey administered during July–August 2017. Participants were recruited from a volunteer online panel by the SSRS survey research firm (ssrs. org). Panel members who resided anywhere in the US were eligible to participate. Participants could take the survey on the computer or mobile device of their choosing. Parents of adolescent participants provided consent for their children to be on the panel and participate in research; adolescent and young adult participants then provided assent and consent, respectively, to participate in this specific survey. The Johns Hopkins Bloomberg School of Public Health Institutional Review Board approved this study’s procedures (Number 00007941).

### Outcomes

Engagement with online tobacco marketing was assessed by asking participants: ‘In the past 6 months, have you done any of the following: [1] visited a tobacco company website, [2] signed up for email alerts about tobacco products, [3] liked or followed a tobacco company’s social media page, [4] read articles online promoting tobacco products, [5] watched a video online promoting tobacco products, or [6] none of the above?’. Participants could select as many types of engagement from this list, as applied to them. These forms of engagement correspond to the leading forms of engagement among adolescents in the nationally representative Population Assessment for Tobacco and Health Study^9^.

Participants who indicated they had engaged in at least one of the behaviors were then asked an open-ended question: ‘In the prior question you said you [insert behavior(s) from prior question]. What are the reasons you engaged with tobacco companies online?’. This open-ended question was asked once and pertained to all of the types of engagement participants indicated.

To analyze these open-ended data, we created a coding framework (Table 1) based on a thematic analysis of the data. We used a parallel approach that allowed us to identify emergent themes, and also allowed for a priori identification of codes (e.g. engaging for discounts, coupons, incentives or contests) based on research documenting aspects of marketing that are appealing to consumers^12,13^. Categories of reasons for engagement included: [1] ad exposure of ambiguous nature (i.e. unclear if exposure was intentional), [2] intentional ad exposure, [3] incidental ad exposure (e.g. YouTube ad that appeared before viewing intended content), [4] curiosity or seeking general knowledge, [5] product appeal, [6] discounts, coupons, incentives, or contests, [7] online content, [8] family or friends, [9] school project or research purposes, [10] understand adverse effects of tobacco use, and [11] engagement with marketing for specific tobacco product brand. The categories of reasons for engagement were not mutually exclusive because the responses provided by participants often fell into multiple categories (i.e. 31% of responses fell into two categories and 5% of responses fell into three categories). Two authors independently coded the reasons; the interrater agreement, measured by Cohen κ, varied between 0.89 and 1.0 among reasons (Appendix Table 1, Supplementary file). When the coders disagreed, they had a discussion to reach consensus.

**Table 1:** Definitions of reasons for engagement with online tobacco marketing^1^

*Reason*	*Definition*	*Exemplar quotes*
Ads, exposure of ambiguous nature	Any response mentioning ads but not specifying nature of exposure	‘It was an ad’ ‘Ads’
Ad, incidental exposure	Respondent indicates they did not intend to watch the ad and/or it was forced upon them	‘It was a pop-up ad that could not be exited that played during a video I was watching that was not related to tobacco’ ‘It was an ad on YouTube that I couldn’t skip so I had to watch it’ ‘I was streaming my anime and one of the ads was a 45s [second] long video about cigarettes [sic]’
Ads, intentional exposure	Respondent indicates they watched the ad on purpose	‘It popped up as an ad and looked interesting. So I watched the promotion then went to the website to check it out’ The ads they showed were quite interesting and intrigued me enough to click on said advertisements to further check out their products’
Curiosity or general knowledge	Response indicating curiosity or seeking to know more, generally	‘Boredom and curiosity’ ‘Just to keep up with the latest news’
Product appeal	Respondent seeking more information about a specific product, looking to buy a product, or interested in a particular aspect of a product	‘Wanted to buy a vape pen’ ‘Curiosity in new flavors’ ‘Deciding between brands’
Discounts, coupons, incentives, or contests	Respondent indicates seeking free and/or reduced product, or being otherwise incentivized to view and/or buy a product	‘My mother and I are both smokers and it is a very expensive habit. I go to the websites for coupons and deals to try and save money for our lifestyle’ ‘At a concert to receive a free koozie I had to sign up for email alerts’
Online content	Any response referencing online content, including unspecified video content, and social content such as Facebook, Instagram, Twitter, and YouTube	‘I liked a Marlboro post and it pops up in my feed’ ‘I see ads on Facebook. My friends like their page so I liked it as well’
Family or friends	Any response referencing family or friends, including seeking reduced products for family/friends or being exposed to online tobacco content by family/friends	‘One of my friends promoted it on Twitter’ ‘Finance [sic] smokes, trying to make them cheaper until she can quit. Trying to switch to e-cigs’
School or research	Respondent specified engagement with online tobacco content was for school project or research purposes	‘It was an ad I had to watch for a class’ ‘Research for a school project’
Adverse effects or anti-tobacco sentiment	Respondent specified engagement with online tobacco content was to understand negative health impact, or admitted bias against tobacco products	‘There are always people who want to quit and don’t know how. I’m not addicted so I try to help’ ‘Because I don’t want to get cancer’ ‘I saw one and wanted to read why people would be promoting tobacco products because it’s revolting’
Particular brand	Respondents indicated a specific brand as their reason for engagement	‘American Spirit was offering $2 packs of [sic] their cigarettes’ ‘I was curious about the Juul’

1 N=5244 US adolescents and young adults sampled in 2017.

Our use of the term engagement is premised upon the hierarchy-of-effects model of persuasion that conceptualizes exposure to a message as distinct from cognitive or affective processing or interaction with the message^14,15^. In this approach, an individual could be exposed to an ad but not register or recall that exposure (e.g. an individual walking on a busy street may not take note of or recall the many ads that are passed by, even though technically exposed to them), and exposure is a necessary pre-requisite to further engagement with the ad (i.e. attending to the message). The study asked participants to recall advertising to which they were exposed. Thus, our study refers to exposure to and engagement with online tobacco marketing as ‘engagement’, since it captures the range of message processing; from merely attending to and recalling an exposure, to more intensive forms of engagement such as actively searching for coupons.

### Covariates

Respondents’ age was categorized as adolescents (13–17 years) and young adults (18–24 years). Gender was categorized as female, male, genderqueer or gender non-confirming, different identity, trans female or trans woman, and trans male or trans man. Race/ethnicity was categorized as non-Hispanic white, Hispanic, non-Hispanic black, non-Hispanic Asian or Pacific Islander, and non-Hispanic American Indian or Alaskan Native.

Respondents were categorized as non-susceptible never tobacco users; susceptible never tobacco users; ever tobacco users, but not within the past 30 days; and past 30-day tobacco users. Never-tobacco-using respondents were considered non-susceptible if they responded ‘definitely not’ to each of the questions: ‘Do you think you will try a (cigarette, e-cigarette or vape pen, cigarillo or filtered cigar, or smokeless tobacco) soon?’, ‘If one of your best friends were to offer you a cigarette, e-cigarette or vape pen, cigarillo or filtered cigar, or smokeless tobacco, would you use it?’, and ‘Do you think you will smoke a cigarette in the next year?’^16^. Never-tobacco-using respondents were considered susceptible if they responded probably not, probably yes, or definitely yes to at least one of these questions. Respondents were considered ever tobacco users if they indicated they had ever tried cigarettes, e-cigarettes, cigarillos/filtered cigars, or smokeless tobacco products. Respondents were considered past 30-day tobacco users if they indicated they had used any of the products in the past 30 days.

### Analysis

First, the prevalence of engagement with online tobacco marketing within the past six months (hereafter, ‘engagement’) was calculated by age group and by tobacco use status. Differences in prevalence by age group were assessed with a t-test for proportions. A difference by tobacco use status was assessed with a one-way analysis of variance and post-hoc pairwise comparisons were performed; p-values were adjusted for multiple comparisons using the Bonferroni method. Second, the prevalence of specific reasons for engagement was calculated by age group and tobacco use status. Differences between age groups and among tobacco use statuses were similarly assessed. Third, multivariable logistic regression models were fit with the following outcomes: [1] any engagement, [2] engagement because of incidental ad exposure, [3] engagement because of curiosity or general knowledge, [4] engagement because of discounts, coupons, incentives, or contests, and [5] engagement because of product appeal. Covariates included: age group, gender, race/ethnicity, and tobacco use status. All analyses performed in R, version 3.5.1^17^.

## RESULTS

### Study population

The sample consisted of 49.9% adolescents and 50.1% young adults (Table 2). The gender distribution was 53.3% female; 45.3% male; 0.8% genderqueer, gender non-conforming, or different identity; 0.6% trans male; and 0.1% trans female. The sample was 59.4% non-Hispanic white, 19.6% Hispanic, and 11.0% non-Hispanic black. Finally, the sample consisted of 25.1% non-susceptible never tobacco users; 30.4% susceptible never tobacco users; 24.1% ever tobacco users, but not within the past 30 days; and 20.3% past 30-day tobacco users.

**Table 2:** Characteristics of sample

	*N*	*Prevalence (%)*
**Age Group**		
Adolescent	2619	49.9
Young adult	2625	50.1
**Gender^1^**		
Female	2794	53.3
Male	2373	45.3
Genderqueer, gender non-conforming, or different identity	40	0.8
Trans male/trans man	33	0.6
Trans female/trans woman	4	0.1
**Race/Ethnicity^1^**		
Non-Hispanic white	3056	59.4
Hispanic	1006	19.6
Non-Hispanic black	564	11.0
Non-Hispanic Asian or Pacific Islander	420	8.2
Non-Hispanic American Indian or Alaskan Native	96	1.9
**Tobacco use status^1^**		
Never tobacco user, not susceptible	1318	25.1
Never tobacco user, susceptible	1595	30.4
Ever tobacco user	1266	24.1
Past 30-day tobacco user	1065	20.3

1 Proportion of respondents do not sum to 100% because of rounding.

### Prevalence of and reasons for engagement with online tobacco marketing

Twelve per cent of adolescents and 28.3% of young adults had engaged with online tobacco marketing within the past six months (Table 3). The leading reason for engagement among adolescents were incidental ad exposure (2.6%), curiosity or general knowledge (2.2%), and online content (1.7%). The leading reasons for engagement among young adults were curiosity or general knowledge (5.5%); incidental ad exposure (5.0%); and discounts, coupons, incentives, or contests (4.5%).

**Table 3:** Prevalence (%) of any engagement with online tobacco marketing within past six months and specific reasons for engagement by age group^1^

	*Adolescents (N=2619 )*	*Young adults (N=2625 )*	*p*
**Any engagement**	12.0	28.3	<0.01
**Specific reasons for engagement^2^**			
Ads (Incidental exposure)	2.6	5.0	<0.01
Curiosity or general knowledge	2.2	5.5	<0.01
Online content	1.7	3.0	<0.01
Discounts, coupons, incentives, or contests	1.3	4.5	<0.01
Product appeal	1.1	3.9	<0.01
Adverse effects or anti-tobacco sentiment	0.8	1.5	<0.01
School or research	0.4	0.9	0.01
Family or friends	0.4	1.4	<0.01
Particular brand	0.4	1.6	<0.01
Ads (Intentional exposure)	0.2	0.4	0.09
Ads (Ambiguous exposure)	0.8	1.7	<0.01

1 N=5244 US adolescents and young adults sampled in 2017.

2 Sum of specific reasons for engagement do not equal the prevalence of any engagement because respondents could indicate multiple reasons.

The prevalence of any engagement varied substantially across tobacco use status: 3.6% for non-susceptible never tobacco users; 15.3% for susceptible never tobacco users; 20.7% for ever tobacco users, but not within the past 30 days; and 47.0% for past 30-day tobacco users (Table 4). Across all tobacco use statuses, the leading reasons for engagement were curiosity or general knowledge (3.9%); incidental ad exposure (3.8%); and discounts, coupons, incentives, or contests (2.9%). Among non-susceptible never tobacco users, the leading reasons for engagement were incidental ad exposure (1.2%), curiosity or general knowledge (0.5%), ambiguous ad exposure (0.4%). Among susceptible never tobacco users, the leading reasons for engagement were incidental ad exposure (4.5%), curiosity or general knowledge (3.1%), and online content (2.3%). Among ever, but not past 30-day tobacco users, the leading reasons for engagement were incidental ad exposure (5.8%), curiosity or general knowledge (4.7%), and online content (3.7%). Finally, among past 30-day tobacco users, the leading reasons for engagement were discounts, coupons, incentives, or contests (11.1%), product appeal (8.9%), and curiosity or general knowledge (8.2%).

**Table 4:** Prevalence (%) of reasons for engagement with online tobacco marketing by tobacco use status^1^

	*Total (N=5244 )*	*Groups*				*p*	*Significantly different groups*
		*1 Never, nonsusceptible (N=1318 )*	*2 Never, susceptible (N=1266 )*	*3 Ever tobacco user, not past 30-day (N=1595 )*	*4 Past 30-day tobacco user (N=1065 )*		
**Any engagement**	20.1	3.6	15.3	20.7	47.0	<0.01	
**Specific reasons for engagement^2^**							1 & 2; 1 & 3, 1 & 4; 2 & 3, 2 & 4; 3 & 4
Curiosity or general knowledge	3.9	0.5	3.1	4.7	8.2	<0.01	1 & 2; 1 & 3, 1 & 4; 2 & 4; 3 & 4
Ads (Incidental exposure)	3.8	1.2	4.5	5.8	3.8	<0.01	1 & 2; 1 & 3, 1 & 4
Discounts, coupons, incentives, or contests	2.9	0.1	0.7	1.7	11.1	<0.01	1 & 2; 1 & 3, 1 & 4
Product appeal	2.5	0.1	0.6	2.1	8.9	<0.01	1 & 3, 1 & 4; 2 & 4; 3 & 4
Online content	2.3	0.4	2.3	3.7	3.2	<0.01	1 & 2; 1 & 3, 1 & 4
Adverse effects or anti-tobacco sentiment	1.2	0.2	1.8	1.4	1.1	<0.01	1 & 2; 1 & 3
Ads (Ambiguous exposure)	1.2	0.4	1.4	1.0	2.2	<0.01	1 & 4
Particular brand	1.0	0.2	0.3	0.6	3.5	<0.01	1 & 2; 1 & 3, 1 & 4
Family or friends	0.9	0.1	1.0	1.2	1.5	<0.01	1 & 3; 1 & 4
School or research	0.6	0.2	0.8	0.7	0.8	0.17	—^3^
Ads (Intentional exposure)	0.3	0.0	0.2	0.6	0.6	0.02	—^3^

1 N=5244 US adolescents and young adults sampled in 2017.

2 Sum of specific reasons for engagement do not equal the prevalence of any engagement because respondents could indicate multiple reasons.

3 Lack of significantly different pairs possible, despite significance of ANOVA, because the comparison between specific pairs of groups used t-tests adjusted for multiple comparisons while the ANOVA assesses difference among group means.

### Correlates of engagement with online tobacco marketing

Young adults were more likely to engage with online tobacco marketing in the past six months than adolescents (AOR=1.98, 95% CI: 1.69–2.33), shown in Table 5. Non-Hispanic blacks and Hispanics were also more likely to engage than non-Hispanic whites (AOR=1.89, 95% CI: 1.50–2.38; and AOR=1.43, 95% CI: 1.18–1.72; respectively). Also, susceptible never tobacco users; ever tobacco users, but not within the past 30 days; and past 30-day tobacco users were all more likely to engage than non-susceptible never tobacco users (e.g. AOR=4.63, 95% CI: 3.35–6.38; for susceptible never tobacco users). Susceptible never tobacco users were more likely to engage because of curiosity or general knowledge than non-susceptible never tobacco users (AOR=6.81, 95% CI: 2.91–15.95). Susceptible never tobacco users were also more likely to engage because of incidental ad exposure than non-susceptible never tobacco users (AOR=3.68, 95% CI: 2.12–6.37). Past 30-day tobacco users were more likely to engage because of discounts, coupons, incentives, or contests and product appeal than ever, but not past 30-day tobacco users (AOR=7.10, 95% CI: 4.41–11.45; and AOR= 4.29, 95% CI: 2.72–6.75; respectively), see Appendix Table 2, Supplementary file.

**Table 5:** Logistic regression of any engagement with online tobacco marketing within the past six months and specific reasons of engagement^1^

	*Any engagement AOR ( 95% CI)*	*Ad, incidental exposure AOR ( 95% CI)*	*Curiosity or general knowledge AOR ( 95% CI)*	*Discounts, coupons, incentives, or contests AOR ( 95% CI)*	*Product appeal AOR ( 95% CI)*
**Young adult** (Ref: Adolescent)	1.98 (1.69–2.33)	1.74 (1.27–2.38)	1.79 (1.29–2.48)	2.06 (1.36–3.13)	1.91 (1.23–2.97)
**Gender** (Ref: Male)					
Female	0.75 (0.65–0.87)	0.86 (0.64–1.15)	0.89 (0.67–1.19)	1.15 (0.82–1.63)	0.60 (0.42–0.88)
Other^2^	0.74 (0.39–1.38)	1.39 (0.54–3.55)	0.92 (0.28–3.02)	0.57 (0.08–4.32)	0.41 (0.05–3.07)
**Race/Ethnicity** (Ref: Non-Hispanic white)					
Non-Hispanic black	1.89 (1.50–2.38)	1.14 (0.70–1.84)	2.24 (1.51–3.32)	0.42 (0.22–0.82)	1.37 (0.79–2.37)
Hispanic	1.43 (1.18–1.72)	1.35 (0.94–1.94)	1.32 (0.92–1.90)	0.42 (0.26–0.70)	1.19 (0.76–1.88)
Non-Hispanic Asian or Pacific Islander	1.81 (1.39–2.37)	1.98 (1.27–3.10)	1.65 (1.00–2.72)	0.62 (0.29–1.32)	1.30 (0.65–2.62)
Non-Hispanic American Indian or Alaskan Native	1.64 (0.98–2.76)	2.45 (1.10–5.47)	0.91 (0.28–2.96)	0.44 (0.10–1.86)	2.56 (1.03–6.38)
**Tobacco use status** (Ref: Non-susceptible never tobacco user)					
Susceptive never tobacco user	4.63 (3.35–6.38)	3.68 (2.12–6.37)	6.81 (2.91–15.95)	9.52 (1.23–73.81)	8.05 (1.03–62.98)
Ever tobacco user, not past 30-day	5.86 (4.24–8.10)	4.22 (2.42–7.35)	9.40 (4.02–21.97)	19.28 (2.58–143.94)	22.78 (3.07–168.74)
Past 30-day tobacco user	19.27 (14.02–26.50)	2.55 (1.40–4.64)	16.09 (6.95–37.22)	136.94 (19.01–986.28)	97.67 (13.53–704.87)

1 N=5244 US adolescents and young adults sampled in 2017.

2 Genderqueer, gender non-conforming, different identity, trans male/trans man, or trans female/trans woman.

AOR: adjusted odds ratio, CI: confidence interval.

## DISCUSSION

This study reports three central findings on the reasons for engagement with online tobacco marketing. First, a substantial proportion of engagement originated from incidental exposure to tobacco ads, especially among adolescents and never tobacco users. Second, online tobacco marketing may enable susceptible never tobacco users to satiate their curiosity about tobacco use and tobacco products. Third, more than one in ten adolescent and young adult past 30-day tobacco users engaged with online tobacco marketing to seek price discounts and coupons for tobacco products.

The high prevalence of incidental exposure to tobacco advertisements online may result in public health harm, especially to non-susceptible and susceptible never tobacco users. A large body of evidence has concluded exposure to tobacco advertising—even brief exposure—in traditional media channels affects adolescents’ perceptions and intentions to smoke and increases the risk of cigarette smoking^12^. Exposure to e-cigarette advertisements may also increase the risk of e-cigarette use among adolescents^18^. Furthermore, exposure to online tobacco advertising and marketing is associated with susceptibility to tobacco use among adolescents and young adults^19^.

Intentional exposure to tobacco marketing also presents a potential public health harm. Youth who are interested in or curious about tobacco use are able to engage with online tobacco marketing to learn more about tobacco products. Moreover, coupons and promotional campaigns lower the cost of tobacco products for users, many of whom are price sensitive^20^, thus promoting the continuation of cigarette smoking. Although relatively few youth report recently receiving tobacco coupons^21^, our study supports evidence that for those who do receive coupons, online channels may be a common source. While tobacco and e-cigarette websites require age verification, the verification process may not be stringent for e-cigarettes or can be bypassed by adolescents for cigarettes^22-24^.

State and local governments could prohibit the redemption of tobacco coupons under their authority to regulate the sale of tobacco products^25,26^. For example, Massachusetts prohibits redemption of coupons for cigarettes that reduce consumers’ retail sale price below a set minimum price^27^. The recent US Supreme Court decision in *South Dakota v. Wayfair Inc., et al.*, now enables states to require internet retailers to collect and remit sales tax^28^. However, while all states impose an excise tax on cigarettes, most states do not impose a similar tax on e-cigarettes^29^.

In addition to state-level regulation, federal regulation can limit engagement with online tobacco marketing. Since 1965, federal regulation has required health warning labels on cigarette packages and advertising (e.g. four rotating Surgeon General’s health warning labels mandated by the Comprehensive Smoking Education Act of 1984). As of August 2018, the Food and Drug Administration (FDA) now requires prominent nicotine addictiveness warning statements on advertisements for all covered tobacco products including e-cigarettes; our study found ad exposure was the leading reason of engagement with online tobacco marketing^30,31^. Previous research has shown prominent warning statements for cigarettes effectively promote smoking cessation and prevent smoking initiation^32-34^. The new FDA requirement on warning statements could further increase cessation and decrease initiation since it applies to online tobacco advertisements and across a broad range of tobacco products. Federal regulation of tobacco-related user-generated content on social networking sites may prove more difficult than regulation of such content produced by the tobacco industry itself because the former is constitutionally protected speech^35^.

Beyond government regulation, social networking sites can reduce engagement with online tobacco marketing by rigorously enforcing existing prohibitions on tobacco advertising. For example, the advertising policy of Facebook (and its subsidiary Instagram) states ‘Ads must not promote the sale or use of tobacco products and related paraphernalia.’^36^. Jackler et al.^37^ found more than 100 leading tobacco brands maintained brand-sponsored pages on Facebook, many of which contained direct purchase links and discount coupons. Yet, few of these brand-sponsored pages were age-gated to restrict access to adolescents. Social networking sites, such as YouTube, could regulate user-generated content by deleting it, if it is harmful or dangerous to adolescents^38^.

### Limitations

We note several important limitations in this analysis. First, although our sample was national, it was not a random sample and thus may not completely reflect the entirety of US adolescents and young adults. Second, given the online modality of the survey, the sample may be skewed in favor of individuals with access to the internet, either on a computer or on a mobile device. However, recent studies estimate that most adolescents and young adults have internet access^39^, and online self-administered survey modalities are useful when asking about sensitive topics such as tobacco use^40^. Third, we also asked participants to reflect upon tobacco marketing with which they engaged during the prior six months. It is possible that this underestimates engagement with tobacco marketing as participants may not recall all tobacco marketing to which they were exposed during this timeframe, or participants may have engaged with tobacco marketing prior to the past six months. Participants were asked if they engaged with online tobacco marketing and the reasons why they engaged, and were not asked the extent to which they found the marketing engaging. Fourth, our study may conservatively estimate the level of engagement with online tobacco marketing because it did not list specific tobacco products (e.g. e-cigarettes) when asking about this behavior. Some respondents may not have considered e-cigarettes to be tobacco products, and thus did not report ways they engaged with e-cigarette marketing. Finally, the study was not sufficiently powered to ascertain differences in the level of engagement of online tobacco marketing among sexual and gender minority populations.

## CONCLUSIONS

Engagement with online tobacco marketing often occurred because of contact with tobacco ads. A substantial proportion of current tobacco users engaged with online tobacco marketing to obtain price discounts and coupons for tobacco products. Stricter state and federal regulation of tobacco marketing and stronger self-regulation by social networking sites could reduce youth engagement with online tobacco marketing. These efforts could reduce the initiation and continuation of tobacco use among adolescents and young adults.

## CONFLICTS OF INTEREST

The authors declare that they have no competing interests, financial or otherwise, related to the current work. Moran reports grants from NIH/FDA, during the conduct of the study. The rest of the authors have also completed and submitted an ICMJE form for disclosure of potential conflicts of interest.
