# Supplementary material for: Reasons for engagement with online tobacco marketing among US adolescents and young adults
Source: Tob Induc Dis. 2019 Jan 10;17:02. doi: 10.18332/tid/99540 (PMC6751987; doi:10.18332/tid/99540)
Supplement: Supplementary file 1 [file TID-17-02-s1.pdf]

Supplemental file: Reasons for Engagement with Online Tobacco Marketing Among US Adolescents and Young Adults

Table 1. Interrater agreement, measured by Cohen κ, among reasons of engagement with online tobacco marketing

Specific reasons for engagement	Cohen κ
Online content	1.00
Ads (Ambiguous exposure)	1.00
Ads (Incidental exposure)	1.00
Particular brand	1.00
Discounts, coupons, incentives, or contests	1.00
Product Appeal	0.99
Family or friends	0.99
School or research	0.99
Curiosity or general knowledge	0.97
Adverse effects or anti-tobacco sentiment	0.95
Ads (Intentional exposure)	0.89

Table 2. Logistic regression (AOR, 95% CI) of any engagement with online tobacco marketing within the past six months and specific reasons of engagement (Reference for tobacco use status is ever tobacco user, not past 30 days)[1]

	Any engagement	Ad, incidental exposure	Curiosity or general knowledge	Discounts, coupons, incentives, or contests	Product appeal
Young Adult (Ref: Adolescent)	1.98 (1.69, 2.33)	1.74 (1.27, 2.38)	1.79 (1.29, 2.48)	2.06 (1.36, 3.13)	1.91 (1.23, 2.97)
Gender (Ref: Male)					
Female	0.75 (0.65, 0.87)	0.86 (0.64, 1.15)	0.89 (0.67, 1.19)	1.15 (0.82, 1.63)	0.60 (0.42, 0.88)
Other[2]	0.74 (0.39, 1.38)	1.39 (0.54, 3.55)	0.92 (0.28, 3.02)	0.57 (0.08, 4.32)	0.41 (0.05, 3.07)
Race/Ethnicity (Ref: Non-Hispanic White)					
Non-Hispanic Black	1.89 (1.50, 2.38)	1.14 (0.70, 1.84)	2.24 (1.51, 3.32)	0.42 (0.22, 0.82)	1.37 (0.79, 2.37)
Hispanic	1.43 (1.18, 1.72)	1.35 (0.94, 1.94)	1.32 (0.92, 1.90)	0.42 (0.26, 0.70)	1.19 (0.76, 1.88)
Non-Hispanic Asian or Pacific Islander	1.81 (1.39, 2.37)	1.98 (1.27, 3.10)	1.65 (1.00, 2.72)	0.62 (0.29, 1.32)	1.30 (0.65, 2.62)
Non-Hispanic American Indian or Alaskan Native	1.64 (0.98, 2.76)	2.45 (1.10, 5.47)	0.91 (0.28, 2.96)	0.44 (0.10, 1.86)	2.56 (1.03, 6.38)
Tobacco Use Status (Ref: Ever Tobacco User, Not Past 30 Days)					
Non-Susceptible Never Tobacco User	0.17 (0.12, 0.24)	0.24 (0.14, 0.41)	0.11 (0.05, 0.25)	0.05 (0.01, 0.39)	0.04 (0.01, 0.33)
Susceptive Never Tobacco User	0.79 (0.65, 0.97)	0.87 (0.62, 1.23)	0.72 (0.49, 1.07)	0.49 (0.24, 1.04)	0.35 (0.17, 0.74)
Past 30-Day Tobacco User	3.29 (2.73, 3.97)	0.60 (0.40, 0.90)	1.71 (1.21, 2.42)	7.10 (4.41, 11.45)	4.29 (2.72, 6.75)

[1]N=5244 US adolescents and young adults sampled in 2017.
[2]Genderqueer, gender non-conforming, different identity, trans male/trans man, or trans female/trans woman.
AOR: adjusted odds ratio. CI: confidence interval.

© 2019 Soneji S.
